# Impact of ribotype on *Clostridioides difficile* diagnostics

**DOI:** 10.1007/s10096-019-03772-z

**Published:** 2019-12-28

**Authors:** Kristina Rizzardi, Thomas Åkerlund, Torbjörn Norén, Andreas Matussek

**Affiliations:** 1 Department of Microbiology, Public Health Agency of Sweden, Solna, Sweden; 2 School of Medical Sciences, Örebro University, Örebro, Sweden; 3 Division of Clinical Microbiology, Department of Laboratory Medicine, Karolinska University Hospital, Huddinge, Sweden; 4 Karolinska University Laboratory, Solna, Stockholm Sweden; 5 Department of Laboratory Medicine, Jönköping, Jönköping County Sweden

## Abstract

This study investigates the performance of diagnostic methods for detection of *Clostridioides difficile* infection in Sweden, including impact of PCR ribotype on diagnostic performance. Between 2011 and 2016, a total of 17,878 stool samples from 26 laboratories were tested by either well-type enzyme immunoassays (EIAs), membrane bound EIAs, cell cytotoxicity neutralization assay (CTA), or nucleic acid amplification tests (NAATs) and subsequently cultured for *C. difficile*. Roughly half of the samples (9454/17878) were subjected to diagnostic testing both on the fecal sample and on the 1323 isolated *C. difficile* strains. All *C. difficile* isolates were typed by PCR ribotyping, and the isolates were classified as toxigenic or non-toxigenic based on the empirical knowledge of the association between toxin-positivity and ribotype. The overall sensitivity, specificity, and positive and negative predictive values were highest for NAATs and membrane EIAs. Ribotype-specific sensitivity varied greatly between methods and ribotypes. All methods had 100% sensitivity against ribotype 027 and 013. For other types, the sensitivity ranged from 33 to 85% in fecal samples and from 78 to 100% on isolates. For the most prevalent ribotypes (014, 020, and 001), the sensitivity varied between 38 and 100% in the fecal samples, with the lowest sensitivity observed for well-type EIAs and CTA. The large variation in diagnostic sensitivity implies that type distribution significantly affects the outcome when evaluating diagnostic performance. Furthermore, performing comparative studies of diagnostic tests in settings with high prevalence of ribotype 027 will overestimate the general performance of diagnostic tests.

## Introduction

*Clostridioides difficile* infection (CDI) is one of the most common healthcare-associated infections worldwide. A European point prevalence survey accredited 48% of all healthcare-associated gastrointestinal infections to CDI with an attributable mortality of 3% [1]. Toxigenic strains of *C. difficile* produce one or two major toxins, enterotoxin (toxin A), and cytotoxin (toxin B) encoded by the pathogenicity locus (PaLoc) [2]. The ability of a strain to produce one or both toxins is crucial for clinical disease. The toxins cause colonic tissue damage to the enteric cytoskeletal wall and disruption of the tight junctions that connect colonic cells [3]. Toxin production is dependent by growth phase, nutritional status and can vary greatly between strains of the same ribotype [4, 5]. Some strains produce also the binary actin-ADP-ribosylating toxin that increases microtubule polymerization which might increase the adherence of *C. difficile* to target cells [6].

In Sweden, around 6000 new cases of CDI are reported yearly, and even though incidence has decreased in recent years [7], it remains a significant burden for the patient and the health care system. An accurate diagnosis of CDI remains a challenge, and underdiagnosis is an issue in Europe [8]. False negative CDI test results may increase the risk of transmission in addition to mistreatment of the patient, while false positive results may lead to unnecessary treatment interventions for CDI. A wide variety of diagnostic tests are available to detect *C. difficile* toxins, i.e., enzyme immunoassays (well-type EIAs), membrane bound enzyme immunoassays (membrane EIA), cell cytotoxicity neutralization assay (CTA), and toxigenic culture (TC), or to detect the toxin genes of *C. difficile* using nucleic acid amplification tests (NAATs). In 2016, the European Society of Clinical Microbiology and Infectious Diseases (ESCMID) published updated guidelines for CDI diagnostics [9], recommending two-step algorithms that aims to detect the presence of the bacteria with a highly sensitive test and free toxins using EIAs. However, the optimal method for the detection of CDI is still under debate [10]. One drawback of many studies comparing diagnostic techniques is that the local epidemiology is not taken into consideration when assessing the performance of a diagnostic test, and several studies have been carried out in high prevalence or outbreak settings. In this study, we aim to assess the ribotype-specific sensitivity of different diagnostic methods in addition to evaluating the overall performance of each method in a non-outbreak setting with high ribotype diversity [7]. The choice of reference method or gold standard is crucial in order to assess the accuracy of a test. In this study, we use PCR ribotyping to determine if an isolate belongs to a toxigenic or non-toxigenic ribotype as gold standard instead of using the standard approach of CTA, toxigenic or cytotoxigenic culture. As the ability of a strain to produce toxins is crucial to diagnose CDI, we do not aim to evaluate the performance of glutamate dehydrogenase tests for the detection of *C. difficile* bacteria in this study.

## Methods

### Sampling, diagnostic testing, and culturing

This prospective study was conducted on a national level in Sweden and included all 26 local laboratories performing diagnostics for *C. difficile*. Sampling was performed twice yearly between 2011 and 2016, during 1 week in spring (w11) and 1 week in autumn (w39). Fecal samples from both hospital- and community-associated cases were included in the study. Eligible patients included those with suspected CDI, for whom unformed stool samples were sent for CDI testing regardless of age of the patient. Information about diagnostic method and/or algorithm and test performance results were registered. Fecal samples were tested at the local laboratory and *C. difficile* isolates were sent to the Public Health Agency of Sweden for PCR ribotyping. Multiple samples from the same patient were allowed due to use of anonymous data.

All laboratories were asked to culture every stool sample tested for CDI during the collection period and to perform CDI diagnostics both on the fecal sample as well as on the cultured isolate using their standard diagnostic test. The diagnostic tests adopted during the collection period were well-type EIAs (VIDAS (bioMeriéux, France)) and Premier Toxin A&B (Meridian bioscience, USA), membrane EIAs (*C. diff* Quik Check Complete (Techlab, USA)), Immunocard Toxin A&B (Meridian bioscience, USA), NAATs (Illumigene (Meridian bioscience, USA)), Gene Xpert (Cepheid, USA), GenomEra® *C. difficile* (Abacus diagnostica, Finland), and CTA. Several laboratories switched methods during the study period, and the tests were performed by different technicians. TCCFA selective media was used for isolation of *C. difficile* [11].

### PCR ribotyping

PCR ribotyping between 2011 and 2012 was performed by a gel-based method according to the method of Stubbs et al. [12] with modifications described in Svenungsson et al. [13] and from 2013 and onwards by capillary gel electrophoresis [14]. The ribotypes classified as non-toxigenic have been described previously in the literature [15, 16].

## Results

**Fig. 1:**
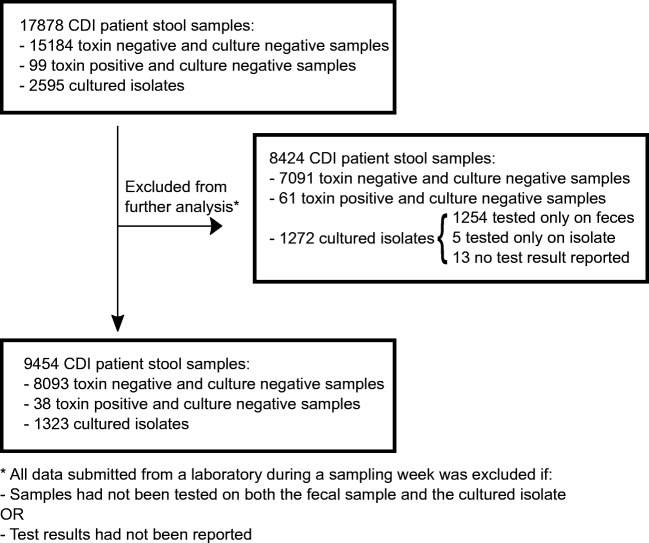
Flowchart of selection process for the samples included in the study

A total of 17,878 stool samples and 2595 *C. difficile* isolates were collected for the study, 99 fecal samples (0.6%) tested positive for the presence on toxins A/B (*n* = 65) or toxigenic *C. difficile* by NAAT (*n* = 34) but were *C. difficile* culture-negative (Fig. 1). Samples were judged to be positive or negative for CDI based on the local laboratories routine interpretation of the test result. Fifty-one percent of the isolates (1323/2595) were tested according to the given instructions i.e. tested both on the fecal sample and on the cultured isolate. For 1254/2595 isolates (48%), only the fecal samples were tested for CDI, and the isolates were never subjected to further testing due to the lack of compliance to the study instructions by the local laboratories. Furthermore, for five isolates, the results from the fecal samples test were not recorded; for 13 isolates, no test results were reported. Samples not tested according to the given instructions and samples with missing information on CDI test results were all excluded from further analysis. Samples reported with a threshold value for CDI, as determined by the manufacturer (VIDAS, bioMeriéux), were considered as negative in this study. Five different types of diagnostic methods or combination of methods were used during the collection period (Table 1).

**Table 1:** CDI testing result from the routine interpretation of clinical laboratories for each *C. difficile* isolate collected according to the study protocol divided by diagnostic method

CDI test result (feces/isolate)	CTA	Well-type EIA	Membrane EIA	NAAT	NAAT + well-type EIA	Total
-/-	11	93		28	1	133
th/-		12				12
-/th		12				12
-/+	28	170	6	37	14	255
th/th		6				6
th/+		14				14
+/-		23		1	1	25
+/+	67	358	33	393	8	859
+/th		7				7
Total	106	695	39	459	24	1323

+, CDI test positive; -, CDI test negative; *th*, threshold/CDI test deemed negative; *CTA*, cell-cytotoxicity neutralization assay; *EIA*, enzyme immunoassay; *NAAT*, nucleic acid amplification test

### Comparison of test performance

A total of 9454/17878 (53%) patient samples were tested according to the given instructions, and 1323 isolates of *C. difficile* were cultured. The majority of samples were analyzed either by well-type EIA (52%) or NAAT (32%), while 11% were analyzed by CTA, 3% by membrane EIA, and 2% by NAAT + well-type EIA. The prevalence of CDI in this study was defined as the number of isolates with a toxigenic ribotype divided by the number of clinical samples tested for CDI and amounted to 13% (1226/9454). The specificity values were high for all methods (> 99%), whereas the sensitivity values varied widely between methods. The NAAT + well-type EIA algorithm showed the lowest sensitivity (41%), while NAAT as a standalone test had the highest sensitivity (91%). Similarly, the lowest negative predictive value (NPV) was reported for NAAT + well-type EIA and the highest for NAAT (Table 2). NAAT + well-type EIA had the highest positive predictive value (PPV), but NAATs and membrane EIAs performed best having both high PPV and NPV.

**Table 2:** Performance of diagnostic methods or algorithm on fecal samples compared with ribotype toxigenicity as the gold standard

	CTA	Well-type EIA	Membrane EIA	NAAT	NAAT + well-type EIA
PPV % (95% CI)	95.7 (95.5–95.9)	93.5 (93.5–93.6)	97.1 (96.9–97.2)	95.1 (95.1–95.2)	100 (100–100)
NPV % (95% CI)	96.5 (96.5–96.5)	94.2 (94.3–94.3)	97.8 (97.7–97.8)	98.5 (98.5–98.5)	92.8 (92.7–92.9)
Specificity % (95% CI)	99.7 (99.7–99.7)	99.4 (99.4–99.4)	99.6 (99.6–99.6)	99.2 (99.2–99.3)	100 (100–100)
Sensitivity % (95% CI)	67.0 (66.7–67.3)	59.7 (59.6–59.8)	84.6 (84.2–85.0)	90.6 (90.6–90.8)	40.9 (40.2–41.6)

*PPV*, positive predictive value; *NPV*, negative predictive value, *CI*, confidence interval, *CTA*, cell cytotoxicity neutralization assay; *EIA*, enzyme immunoassay; *NAAT*, nucleic acid amplification test

### Ribotype-specific sensitivity

Results from PCR ribotyping was used to determine if an isolate belonged to a toxigenic or non-toxigenic ribotype. A total of 144 different PCR ribotypes were determined, and the 10 most common were 014 (*n* = 141), 020 (*n* = 102), 001 (*n* = 84), 002 (*n* = 84), 078/126 (*n* = 65), 023 (*n* = 59), 005 (*n* = 56), 220 (*n* = 44), 010 (*n* = 43), and 045 (*n* = 43), respectively (other ribotypes *n* = 645).

Seven percent (97/1323) of the isolates belonged to non-toxigenic PCR ribotypes such as 010, 009, 031, 032, 039, and 084. These were predominantly found among the isolates that tested negative in all tests, but 1% (*n* = 13) were positive in feces (Table 3). Toxin negativity by the absence of PaLoc locus was confirmed by whole genome sequencing of 6 arbitrarily chosen isolates of ribotype 010 that tested positive for CDI (data not shown).

**Table 3:** CDI testing results per diagnostic method for 97 isolates belonging to 10 toxin-negative ribotypes, as judged from the routine interpretation at the clinical laboratories

CDI test result (feces/isolate)	CTA	Well-type EIA	NAAT	NAAT + well-type EIA
-/-	2	39	18	1
-/+	4	9	7	1
+/-		4		
+/+		3	4	
+/th		2		
th/-		3		

+, CDI test positive; -, CDI test negative; *th*, threshold/CDI test negative; *CTA*, cell cytotoxicity neutralization assay; *EIA*, enzyme immunoassay; *NAAT*, nucleic acid amplification test

Of the 1226 isolates belonging to toxigenic ribotypes, only isolates of ribotype 027 and 013 had a sensitivity of 100% for all CDI tests on fecal samples. For other types, the overall sensitivity ranged from 33 to 85% in fecal samples and from 78 to 100% on isolates. The ribotype-specific sensitivity for each type of diagnostic test is reported in Table 4. No significant difference was found when comparing ribotype distribution between samples tested by NAAT or well-type EIAs (*p* = 0.98 chi-squared test).

**Table 4:** Ribotype-specific sensitivity for each diagnostic method tested on fecal samples and cultured isolates. Only toxigenic ribotypes are included. Ribotypes are sorted by prevalence in descending order and ribotypes with less than 6 isolates are grouped together

Ribotype	Overall sensitivity feces/isolate (*n*)	CTA sensitivity feces/isolate^b^ (*n*)	Well-type EIA sensitivity feces/isolate^a^ (*n*)	Membrane EIA sensitivity feces/isolate^a^ (*n*)	NAAT sensitivity feces/isolate (*n*)	NAAT + well-type EIA sensitivity feces/isolate (*n*)
014*^ǂ^	77/92 (141)	64/91 (11)	64/86 (72)	100/100 (2)	98/100 (55)	0/100 (1)
020*^ǂ^	65/89 (102)	38/88 (8)	48/82 (56)	100/100 (1)	95/100 (37)	ND
001*	76/95 (84)	64/100 (11)	67/90 (39)	83/100 (6)	93/100 (28)	ND
002*^ǂ^	77/88 (84)	71/86 (7)	68/78 (41)	100/100 (4)	90/100 (30)	50/100 (2)
078/126*^ǂ^	62/80 (65)	100/100 (6)	38/67 (39)	100/100 (2)	94/100 (18)	ND
023*^ǂ^	78/88 (59)	67/100 (3)	68/79 (34)	100/100 (1)	100/100 (20)	0/100 (1)
005*	66/93 (56)	33/67 (3)	57/89 (28)	67/100 (3)	89/100 (19)	33/100 (3)
220*	50/86 (44)	50/100 (2)	40/80 (30)	60/100 (5)	100/100 (6)	0/100 (1)
045*	77/86 (43)	100/100 (4)	62/81 (26)	100/100 (1)	100/100 (11)	100/0 (1)
029	65/95 (40)	100/100 (1)	57/90 (21)	100/100 (2)	79/100 (14)	0/100 (2)
081	85/94 (34)	100/100 (2)	50/94 (16)	ND	80/93 (15)	0/100 (1)
012	76/100 (34)	0/100 (2)	80/100 (10)	ND	80/100 (20)	100/100 (2)
070	82/100 (29)	67/100 (3)	77/100 (13)	ND	92/100 (13)	ND
046	59/93 (27)	25/100 (4)	61/89 (18)	ND	80/100 (5)	ND
017	80/88 (25)	ND	72/83 (18)	ND	100/100 (6)	100/100 (1)
018	80/100 (25)	100/100 (4)	64/100 (14)	ND	100/100 (7)	ND
011	79/96 (24)	100/100 (2)	67/92 (12)	ND	89/100 (9)	100/100 (1)
003	61/91 (23)	0/100 (1)	60/87 (15)	0/100 (2)	100/100 (5)	ND
026*	65/85 (20)	100/100 (2)	17/83 (6)	100/100 (1)	82/82 (11)	ND
570	69/88 (16)	100/100 (1)	63/100 (8)	100/100 (1)	67/67 (6)	ND
231	83/83 (12)	100/100 (1)	67/33 (3)	ND	88/100 (8)	ND
054	70/100 (10)	50/100 (2)	0/100 (2)	ND	100/100 (6)	ND
043	70/90 (10)	100/100 (1)	50/83 (6)	ND	100/100 (3)	ND
258	67/100 (9)	100/100 (1)	67/100 (3)	ND	75/100 (4)	0/100 (1)
027	100/100 (8)	100/100 (1)	100/100 (2)	100/100 (1)	100/100 (4)	ND
103	75/100 (8)	100/100 (1)	33/100 (3)	ND	100/100 (3)	100/100 (1)
015	75/88 (8)	ND	75/75 (4)	100/100 (1)	67/100 (3)	ND
013	100/100 (8)	100/100 (2)	100/100 (4)	ND	100/100 (2)	ND
087	71/100 (7)	ND	75/100 (4)	ND	67/100 (3)	ND
808	33/83 (6)	100/100 (1)	0/67 (3)	ND	50/100 (2)	ND
207	67/83 (6)	ND	67/67 (3)	ND	67/100 (3)	ND
Others	73/81 (159)	53/62 (13)	66/76 (82)	100/100 (6)	89/89 (54)	25/100 (4)
< 6*^ǂ^						
Total*ǂ	68/90 (1226)	67/90 (100)	60/83 (635)	85/100 (39)	91/97 (430)	41/95 (22)

^a^Equivalent to toxigenic culture

^b^Equivalent to cytotoxigenic culture

*Difference between well-type EIA and NAAT on feces is statistically significant at *p* < 0.05 (Fisher’s exact test)

^ǂ^Difference between well-type EIA and NAAT on isolates is statistically significant at *p* < 0.05 (Fisher’s exact test)

*ND*, no data; *CTA*, cell cytotoxicity neutralization assay; *EIA*, enzyme immunoassay; *NAAT*, nucleic acid amplification test

Six isolates of PCR ribotype 023 and two of ribotype 231 that tested negative in feces and/or on the isolate by well-type EIA were sequenced by WGS to determine the presence of toxin genes. All eight isolates carried genes for toxin A, toxin B, and the isolates of ribotype 023 carried also binary toxin genes (data not shown).

## Discussion

A rapid and correct identification of a CDI case is crucial to ensure correct treatment and adequate infection control measures [17]. Reference assays for CDI have been CTA and/or TC; at times, also cytotoxigenic culture has been adopted. The major drawback of these methods is the long turnaround time, and thus they are rarely used in routine diagnostics [18]. All methods have their limit of detection and fecal samples are complex and can inhibit both antibody binding as well as nucleic acid amplification. Furthermore, a drawback of all antibody-dependent tests is the variation in affinity towards all genetic variants of toxin A and/or B, as mutations that change the antibody’s antigen binding site could cause reduced antibody affinity [19]. Similarly, if mutations should arise in the primer regions of toxin genes targeted by NAATs, this could also reduce NAAT sensitivity. By using the toxigenic status associated with a given PCR ribotype as a confirmatory test, we aimed to circumvent the problems caused by limit of detection dependent on mutations that affect affinity or low toxin production caused by nutritional status or growth phase [4, 5]. Some of the toxigenic ribotypes could have lost the ability to produce toxins by homologous recombination of the PaLoc [20], but in such a case, we would observe no difference when comparing the ribotype-specific sensitivity of the fecal samples to the ribotype-specific sensitivity of cultured isolates. Instead, the type-specific sensitivity was higher on isolates compared with fecal samples, irrespectively of diagnostic method or ribotype. By sequencing a subset of isolates, we confirmed that isolates deemed as false negatives actually carried the toxin genes and, vice versa, isolates considered as false positives lacked the toxin genes. As we observed an increased sensitivity when testing on cultured isolates, the major cause of the low sensitivity observed for well-type EIAs and CTA was most likely due to the low amount and/or degradation of toxins in stool samples.

Toxin-negative ribotypes could also have been erroneously isolated from six toxin-positive fecal samples, but this aspect was not investigated further.

By using the toxigenic properties of a ribotype as a gold standard, there is a risk of overestimating CDI prevalence, but the CDI prevalence in this study was similar to that in another Swedish study carried out during the same time period with CTA as a comparative test (prevalence 13.2% [21]). Although this was a small regional study, we believe that comparing performance against the toxigenic properties of a ribotype does not largely affect CDI prevalence.

Enrichment prior to culturing could have improved the results from this study, but the clinical relevance of samples with *C. difficile* concentration too low for culturing is uncertain. Preferably, a new sample should be taken for re-testing if CDI symptoms persist. In this study, all samples were included, thus, also new samples from patients that initially have tested negative.

The results showed that membrane EIAs and NAATs as standalone tests performed best with highest PPV and NPV. However, since only a small number of samples and fewer ribotypes were tested with membrane EIAs, these results should be interpreted carefully. NAAT + well-type EIA had a PPV of 100%, but due to the low sample size, just one false positive result would lower the PPV to 90%. Compared with the pooled values from the ESCMID diagnostic guidance document [9], the NAAT tests in this study had a lower sensitivity but higher specificity. The lower sensitivity may be due to the relatively high ribotype diversity in this study. Many comparative diagnostic studies have been conducted in settings with high prevalence of ribotype 027, which would have resulted in an overestimation of the true performance of a diagnostic test. The higher specificity of NAATs in this study may be due to the inclusion algorithm for patients, i.e. testing only samples based on clinical suspicion for CDI and not all diarrheal samples. Performance outcome may also be affected by the choice of comparative standard method. In this study, the classical gold standard methods, CTA, TC (testing isolates with well-type EIA), and cytotoxigenic culture (testing isolates with CTA), had a sensitivity of 67%, 83%, and 90%, respectively, and all were less sensitive than NAAT performed on feces (91% sensitivity). The poorest sensitivity for CTA and well-type EIAs was observed for some of the most common ribotypes circulating in Sweden, e.g., 014, 020, 078/126, and 005. If cytotoxigenic culture or TC (with well-type EIA) would have been used as gold standard in this study, 12% and 18% of these ribotypes, respectively, would have been reported as false positives using NAAT. The reason for the reduced sensitivity of diagnostic tests that detect toxin A and/or B against the above mentioned ribotypes may be due to the variation in toxin production. Some hypervirulent ribotypes like 027 are known to have higher toxin levels in vitro compared with other common ribotypes [22]. It is possible that toxin production in vivo may vary substantially between strains or during an ongoing infection. Furthermore, immunocompromised patients could develop CDI symptoms also when toxin levels are below the limit of detection.

Using the toxigenic status of a ribotype as a gold standard for diagnosing CDI may have some drawbacks. For example, there is still a possibility that mutations in regulatory elements or within the PaLoc can affect the ability to produce toxins. However, as we observed a higher sensitivity for tests made directly on the isolate, we believe that this is unlikely for the majority of ribotypes. This approach improves the limit of detection compared with traditional gold standard methods but is still not optimal as a gold standard for CDI diagnostics. Possibly, the gold standard for CDI diagnostic test performance would include a test result related to symptom resolution following CDI treatment, thus enabling to diagnose cases where clinical suspicion of CDI is strong despite a negative CDI test.

Although the sample size of ribotype 027 isolates was rather small (*n* = 8, 0.6%), our results indicate a higher performance for all diagnostic methods towards ribotype 027. Thus, caution should be made when interpreting comparative studies of diagnostic tests in settings with high prevalence of ribotype 027. To date, only one study has addressed the effect of strain type on diagnostic sensitivity, and in accordance with the results in this study, NAATs showed higher overall sensitivity compared with EIAs but similar performance against ribotype 027 [23].

Local recommendations for *C. difficile* diagnostic tests should be adapted to ensure the best performance according to local *C. difficile* epidemiology, and take into consideration sample logistics, unless refrigerated, samples should be tested within 2 h from sampling if the test targets free toxins to avoid degradation of toxins [24]. When using NAAT as a standalone test, it is preferred to select stool samples for CDI based on clinical suspicion of CDI to reduce the risk of detecting asymptomatic carriage. Although, when analyzing samples only based on clinical suspicion, there is a risk of underdiagnosis, but this has been shown not to be an issue in Sweden [8], where the current national recommendation is to test only unformed stool samples upon clinical suspicion [25].
